# Efficient native strains of rhizobia improved nodulation and productivity of French bean (*Phaseolus vulgaris* L.) under rainfed condition

**DOI:** 10.3389/fpls.2022.1048696

**Published:** 2022-12-15

**Authors:** Puthan Purayil Athul, Ranjan Kumar Patra, Debadatta Sethi, Narayan Panda, Sujit Kumar Mukhi, Kshitipati Padhan, Sanjib Kumar Sahoo, Tapas Ranjan Sahoo, Satyabrata Mangaraj, Shriram Ratan Pradhan, Sushanta Kumar Pattanayak

**Affiliations:** ^1^ Department of Soil Science and Agricultural Chemistry, College of Agriculture, Odisha University of Agriculture and Technology, Bhubaneswar, Odisha, India; ^2^ Department of Agronomy, College of Agriculture, Odisha University of Agriculture and Technology, Bhubaneswar, Odisha, India; ^3^ Department of Vegetable Science, College of Agriculture, Odisha University of Agriculture and Technology, Bhubaneswar, Odisha, India

**Keywords:** liming, French beans, rhizobium, root nodules, pod yield, boron

## Abstract

Biological nitrogen fixation is the most important eco-friendly approach to nitrogenous fertilizer management in the rhizosphere. *Rhizobium* is considered the most important symbiotic N-fixing microorganism. Native strains of *Rhizobium* perform better than the non-native strains by getting ambient conditions for growth and proliferation. Native strains enhance the soil fertility and productivity of pulses. The study was carried out in three phases, i.e., pot experiment, field experiment, and farmers’ field demonstrations. In a pot experiment, two isolated rhizobia were inoculated to seeds of French bean (*Phaseolus vulgaris*) and applied with and without lime to evaluate crop growth, photosynthetic activity, and nodule characteristics of the target crop. In the field, strains were inoculated to seeds of French bean, which received different combinations of inputs— inorganic fertilizers, lime, and boron— to study the influence of native stains on crop productivity and agronomic efficiency. In comparison to non-limed packaging, the amounts of chlorophyll *a*, chlorophyll *b*, total chlorophyll, and chlorophyll *a*:*b* were, respectively, 13% to 30%, 1% to 15%, 10% to 27%, and 1% to 20% greater in limed packages. In limed packages compared to non-limed packages, the root length, biomass, density, and growth rate were increased by 16% to 17%, 36% to 52%, 38% to 49%, and 36% to 52%, respectively. In contrast to non-limed packages, limed packages had nodule attributes like the number of nodules per plant and nodular weight, which were 28% to 41% and 33% to 37% greater, respectively. Inoculation of native rhizobia strains with liming to acid soil increased 46% to 72% of leaf nitrogen content over non-limed rhizobia inoculated packages. In a field experiment, the adoption of soil test-based fertilizer application had an advantage of 25% in pod yield over farmers’ practice. Acid soil amelioration with lime improved pod yield from 14% to 39% over non-limed packages. Farmers’ field demonstration recorded the highest pod yield in the package where seeds were inoculated with S2 (RBHR-21) strain added with soil test-based fertilizers (STD) followed by STD + S1 (RBHR-15) with 98% and 84% increase over farmers’ practice. However, experimental evidence favored using both strains for bio-inoculation of the French bean crop.

## Introduction

The green revolution has been biased toward cereal production, whereas pulses are the source of staple food, and the protein requirements of human beings have not been given adequate attention. Pulses also contain essential vitamins and minerals required for human health, in addition to other important bioactive compounds (Pattanayak and Rao, 2016). Among food legumes, the French bean (*Phaseolus vulgaris* L.) has been the third most important crop worldwide, superseded by soybean and peanut. In the Kandhamal district of Odisha, the tribal people are cultivating one local cultivar of French bean known as “Raikia bean” during the rainy season as a *rainfed* crop in hilly regions without much cultural practice.

Nitrogen is arguably the most important nutrient required by plants, being an essential component of all proteins, nucleic acids, and other nitrogen compounds. However, nitrogen availability is limited in many soils, although the Earth’s atmosphere consists of 78.1% nitrogen gas (N_2_). In this context, pulses play a significant role in nitrogen cycling, as they fix atmospheric N_2_ through biological processes. The biological nitrogen fixation (BNF) process is a valuable alternative to nitrogen fertilizer. The process of BNF performed by symbiotic nitrogen-fixing bacteria with legume species, commonly known as α and β rhizobia, provides high sustainability for ecosystems (Bomfeti et al., 2011). Leguminous plants that fix N_2_ will grow well on soils that are poor in nitrogen (Pattanayak et al., 2007), thus making it unnecessary to add expensive nitrogenous fertilizers. For this reason, BNF is the most “environment-friendly” approach to supplying N to agroecosystems. Inoculation of rhizobial strain increases the plant growth, nodular properties (Khuntia et al., 2022), and photosynthetic activity of the legumes (Ochieno et al., 2021).

Rhizobia are inoculated in legume crops to enhance biological N-fixation to meet nitrogen requirements and restore soil fertility (Saeed et al., 2021; Rafique et al., 2022). Efficient N-fixation symbiosis between rhizobia and legume is a success of symbiosis relationships. However, several agricultural soils are devoid of an adequate population of rhizobia. The use of efficient rhizobial strains is an important means to combine their significance in improving crop yield and biological control of plant diseases and pests (Yadav et al., 2021).

Legume seed, inoculated with *Rhizobium* culture, increased the crop yield from 20% to 80% and had a beneficial effect on the subsequent crop yield (Lalitha and Immanuel, 2013). Plant growth-promoting bacteria (PGPB) stimulate plant growth both directly and indirectly, therefore establishing a healthy ecological relationship between PGPB and plants (Jiménez-Mejía et al., 2022). The application of PGPB is now considered one of the technologies for the development of sustainable agriculture (Santoyo et al., 2021; Liu et al., 2022).

Utilization of native *Rhizobium* as inoculants promotes the ecologically sustainable management of the agricultural ecosystem and enhances legume production due to their growth-promoting traits and adaptability to soil and environmental stress (Mwangi et al., 2011). Native strains of *Rhizobium* also produce exopolysaccharides, which help in sustaining the roots under drought condition (Sethi et al., 2019b) and also helps in enhancing the soil’s biological activity by improving microbial population and enzyme activity (Sethi et al., 2019a). Amelioration of problematic soil like acidic soil enhances the efficiency of inoculated strains (Sethi et al., 2021). Co-inoculation of native strains with plant growth-promoting rhizobacteria (PGPR) elevates the performance in terms of growth, photosynthetic activity, and nutrient uptake (Sethi et al., 2021). Rhizobia inoculation to the rhizosphere helps to sustain abiotic stress conditions by producing exopolysaccharides and phytohormones (Sethi et al., 2019b; Subudhi et al., 2020; Liu et al., 2022). Furthermore, crop production using inoculants could be cheaper and more affordable to the resource-poor smallholder farmers (Pattanayak, 2016; Pattanayak and Rao, 2014). The ability of native strains to interact positively with the resident soil microbiota and their adaptability to the local agro-ecological climatic conditions often elucidates their superior performance over the exotic commercial strains (Meghvansi et al., 2010; Sethi et al., 2019a). Keeping the above facts in view, two local strains of *Rhizobium* were isolated from French bean nodules collected from the Kandhamal district of Odisha and utilized as biofertilizers for enhancing productivity.

## Materials and methods

### Experimental details

Experiments were carried out in three stages, i.e., infectivity test with the help of micro pot experiment to study the nodulation ability of the test strains of rhizobia in the Department of Soil Science and Agricultural Chemistry, OUAT, Bhubaneswar; field efficiency testing through a field experiment conducted in the research farm of Krishi Vigyan Kendra, Kandhamal during *Rabi* 2019–2020; and the farmers’ field demonstrations undertaken in 15 farmers filed in Kandhamal district. The efficiency of the strains was compared against the local farmers’ practice.

### Sources of rhizobia

Two rhizobia strains were isolated and maintained in the Department of Soil Science and Agricultural Chemistry, College of Agriculture, Odisha University of Agriculture and Technology, Bhubaneswar. National Center for Biotechnology Information (NCBI) accession number of strain RBHR-15 (*Rhizobium etli*) and RBHR-21 (*Rhizobium pisi*) were MN480514 and MN480516, respectively (Verma et al., 2022).

### Pot experiment

The infectivity test was carried out with fine-grained river sand, soil, and vermicompost mixture (1:1:1) sterilized in an autoclave at 20 pounds per square inch (psi) pressure, for 2 h. Half of the soil mixture was treated with lime @ 0.2 lime requirement (LR). Limed and unlimed soil mixtures were then filled in plastic pots of 500-g capacity. Seeds were inoculated with the locally isolated native *Rhizobium* strains and sown in the pots according to the treatments, viz., T_1_, control (no seed inoculation); T_2_, inoculation with *Rhizobium* strain 1 (RBHR-15); T_3_, inoculation with *Rhizobium* strain 2 (RBHR-21); T_4_, liming; T_5_, inoculation with RBHR-15 + Liming; and T_6_, inoculation with RBHR-21 + liming. Treatments were replicated four times. After 7 days, three healthy plants were maintained in each pot by thinning out extra seedlings. Watering was performed by measuring the weight of pots to keep 60% moisture by providing N-free McKnight’s seedling nutrient solution. Observations on nodulation and other parameters were recorded 20, 40, and 60 days after germination (DAG) through destructive sampling.

### Root and nodular properties, leaf nitrogen content, growth rate, and photosynthetic activity

The length of the root was measured by a centimeter scale, and the volume of the root was measured by immersing it in a calibrated measuring cylinder filled with water; the volume (cm^3^) of the displaced water was recorded as root volume. Biomass was recorded after drying in a hot air oven till a constant weight was achieved. Root density was calculated by dividing root biomass (g) by root volume (cm^3^). The total number of nodules was expressed as the number of nodules per plant on a counting basis. Nodular and leaf N were estimated by the Kjeldahl digestion method as described in the Association of Official Analytical Chemists AOAC (1960). Plant height was measured at 20, 40, and 60 DAG and expressed in cm. The growth rate was calculated as the increase in height divided by days interval and expressed as cm day^−1^. Leaf chlorophyll content (*a*, *b*, and total) was estimated and calculated (as per the formula given below) as described by Sadasivam and Manickam (2008).


$$\begin{array}{l} \text{Chlorophyll}-a\;({\text{mg g}}^{-1\;}\text{tissue})\;=12.7\;\left(A_{663\;}\right)-2.69\;\left(A_{645\;}\right)\;\\ \times \;\frac{V}{100\;\times \;W}\;\\ \text{Chlorophyll}-b\;({\text{mg g}}^{-1\;}\text{tissue})=22.9\;\left(A_{645\;}\right)-4.68\;\left(A_{633\;}\right)\;\times \;\frac{V}{100\;\times \;W} \end{array}$$

$$\text{Total chlorophyll }({\text{mg g}}^{-1\;}\text{tissue})\;=20.2\;\left(A_{645\;}\right)-8.02\;\left(A_{633\;}\right)\;\times \;\frac{V}{100\;\times \;W}$$

### Soil analysis

Electrical conductivity and pH of soil samples were determined in 1:2.5 soil: water suspension using a glass electrode digital pH meter and conductivity meter, respectively (Jackson, 1973). The organic carbon content of the soil was determined by modifying Walkley and Black’s rapid titration method (Page et al., 1982). Available nitrogen of soil samples was determined by the alkaline permanganate method as outlined by Subbiah and Asija (1956), Bray’s 1- P was determined by Brays and Kurtz as described by Page et al. (1982), ammonium acetate extractable K as described by Jackson (1973), and available sulfur by turbidometric method (Chesnin and Yien, 1950). Diethylenetriamine pentaacetate (DTPA) extractable Zn was determined by following the method described by Lindsay and Norvell (1978) and estimated by atomic absorption spectrophotometry (AAS). Available boron was determined by the hot water extraction method as outlined by John et al. (1975).

### Field experiment

In the field experiment, two native isolated stains were inoculated to the seeds, and inputs like lime and soil test doses of fertilizers (N:P_2_O_5_:K_2_O: 25:50:40 kg ha^−1^) were applied in different treatment combinations and compared against farmers’ practices. The different treatment combinations were T_1_, absolute control; T_2_, strain-1(S1; RBHR-15); T_3_, strain-2 (S2; RBHR-21); T_4_, framers’ practice (FP; 50:60:25 kg ha^−1^ N: P_2_O_5_: K_2_O, no lime, no B, no balanced fertilizers, and no seed inoculation); T_5_, FP + S1; T_6_, FP + S2; T_7_, FP + S1 + lime @ 0.2LR (L); T_8_, FP + S2 + L; T_9_, soil test dose (STD); T_10_, STD + S1; T_11_, STD + S2; T_12_, STD + S1 + L; T_13_, STD + S2 + L; T_14_, STD + S1 + L + Boron @ 10 kg ha^−1^ (B); and T_15_, STD + S2 + L + B. The treatments were replicated thrice. Relative agronomic efficiency and % yield responses were calculated by using the following formulae (Devasenapathy et al., 2008):


$$\text{Relative agronomic efficiency }\left(\text{RAE}\right)\left(\%\right)=\frac{\text{Fresh pod yield in treatment }-\text{Fresh pod yield in control }}{\text{Fresh pod yield in STD}-\text{Fresh pod yield in control}}\times 100$$

$$\text{Yield response }\left(\%\right)=\frac{\text{Fresh pod yield in treatment }-\text{Fresh pod yield in control }}{\text{Fresh pod yield in treatment}}\times 100$$

### Demonstrations in farmers’ fields

Fifteen farmers were selected for the demonstration. Initial soils were analyzed, and the soil test dose was fixed according to the test values. There were six treatments, viz., farmers’ practice (FP), FP + S1, FP + S2, STD, STD + S1, and STD + S2. The yield of the pod was recorded after harvest.

### Climatic condition

The experimental sites experienced a subtropical climate wherein summers were hot and dry. The monsoon usually started from the second week of June to mid of September. Winter was the longest season and had a dry and cold climate. The average maximum temperature in the district is 45.5° C, and the minimum temperature is 2.0° C. The average annual rainfall recorded is 1,522.95 mm.

### Statistical analysis

The completely randomized design (CRD) was followed for the pot experiment and the randomized block design (RBD) for the field experiment and farmers’ field demonstration. The data generated were analyzed by using the software SPSS 25 and software GRAPES (Gopinath et al., 2020).

## Results

### Pot experiment

#### Crop growth

**Figure 1:**
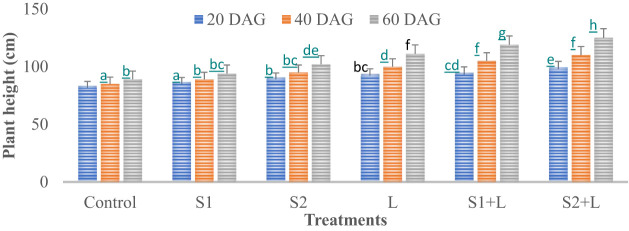
Influence of *Rhizobium* strains inoculation on plant height of French bean. T-shaped bar at the top of each bar indicates standard error (SE), and different letters on top indicate statistical difference (Duncan’s multiple-test range), p = 0.05.

**Figure 2:**
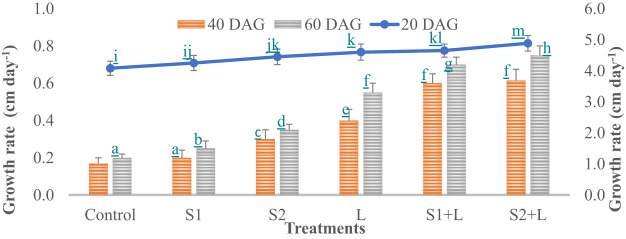
Influence of *Rhizobium* strains inoculation on growth rate of French bean. T-shaped bar at the top of each bar indicates standard error (SE), and different letters on top indicate statistical difference (Duncan’s multiple-test range), p = 0.05.

Data relating to crop growth, viz., plant height (cm) and growth rate, are presented in **Figures 1**, **2**, respectively. Plant height at 20 DAG varied between 81.6 and 97.7 cm with a growth rate of 4.1 to 4.9 cm day^−1^, respectively, depending upon the treatments imposed. Plant height was less under the control treatment, increased considerably (4.2%) due to inoculation with the S1 strain, and almost doubled (9.1%) due to the S2 strain. Liming of soil increased the crop growth by 13.0%, while integrating liming practice with the seed inoculation process increased the crop growth rate to 4.7 and 4.9 cm day^−1^ with the S1 and S2 strains, respectively. Crop achieved a height of 93.0 and 97.7 cm when inoculated with the S1 and S2 strains, respectively, supplemented with liming. The poor crop growth rate was recorded at 40 DAG and 60 DAG in comparison to 20 DAG, due to reduced soil nutrient- supplying ability over time. The growth rate at 40 DAG varied between 0.17 and 0.62 cm day^−1^ with the lowest under control and the highest under L + S2 treatment, even though plant height increased and varied between 85 and 110 cm. At 60 DAG, the growth rate declined, ranging from 0.15 to 0.56 cm day^−1^, and the crop achieved a height between 88 and 121 cm. Seed inoculation had a positive impact on crop growth, specifically with the S2 strain.

#### Photosynthetic activity

The photosynthetic activity of the crop was measured as chlorophyll *a*, chlorophyll *b*, and total chlorophyll content. Data related to the chlorophyll content of the leaf increased from 20 DAG to 60 DAG irrespective of the treatments received and are presented in **Table 1**. Chlorophyll *a* contents at 20, 40, and 60 DAG ranged from 1.1 to 2.8, 1.2 to 3.5, and 1.2 to 4.7 mg g^−1^, respectively. There was an increase in chlorophyll *a* with the native strains’ inoculation and with the combination of lime and sole lime treatments over control. There was an increase in chlorophyll *a* till 60 DAG irrespective of the treatments, whereas in control, it remained almost constant between 40 and 60 DAG (1.20 mg g^−1^). It was due to a poor supply of nitrogen to plants in the control treatment.

**Table 1:** Influence of *Rhizobium* strains on photosynthetic activity of French bean crop.

Treatments	Days after germination (DAG)		
	20	40	60
Chlorophyll *a* (mg g^−1^ fresh leaves)			
Control	1.10 ± 0.02^a^	1.20 ± 0.02^a^	1.20 ± 0.01^a^
Strain*-* 1 (S_1_)	1.70 ± 0.03^b^	2.00 ± 0.04^b^	3.00 ± 0.03^c^
Strain*-* 2 (S_2_)	2.20 ± 0.05^cd^	2.30 ± 0.04^b^	3.60 ± 0.04^d^
Lime (L)	1.80 ± 0.04^bc^	2.10 ± 0.03^b^	2.50 ± 0.03^b^
S_1_ + L	2.40 ± 0.05^cd^	2.90 ± 0.05^c^	3.40 ± 0.04^e^
S_2_ + L	2.80 ± 0.06^d^	3.50 ± 0.06^d^	4.70 ± 0.05^d^
Chlorophyll *b* (mg g^−1^ fresh leaves)			
Control	0.52 ± 0.01^a^	0.60 ± 0.01^a^	0.61 ± 0.02^a^
Strain*-* 1 (S_1_)	0.70 ± 0.02^ab^	0.75 ± 0.02^b^	1.04 ± 0.03^b^
Strain*-* 2 (S_2_)	0.76 ± 0.02^bc^	0.78 ± 0.02^b^	1.20 ± 0.04^c^
Lime (L)	0.70 ± 0.02^ab^	0.84 ± 0.03^cb^	1.00 ± 0.02^b^
S_1_ + L	0.82 ± 0.03^bc^	0.92 ± 0.01^dc^	1.05 ± 0.02^b^
S_2_ + L	0.90 ± 0.04^c^	1.08 ± 0.03^e^	1.39 ± 0.03^d^
Total chlorophyll (mg g^−1^ fresh leaves)			
Control	1.64 ± 0.02^a^	1.83 ± 0.03^a^	1.83 ± 0.03^a^
Strain*-* 1 (S_1_)	2.43 ± 0.03^b^	2.78 ± 0.04^b^	4.08 ± 0.05^c^
Strain*-* 2 (S_2_)	2.97 ± 0.04^c^	3.11 ± 0.06^d^	4.83 ± 0.06^d^
Lime (L)	2.53 ± 0.03^b^	2.96 ± 0.05^c^	3.53 ± 0.04^b^
S_1_ + L	3.26 ± 0.06^d^	3.85 ± 0.06^e^	4.49 ± 0.06^c^
S_2_ + L	3.74 ± 0.06^e^	4.60 ± 0.06^f^	6.12 ± 0.06^e^
Chlorophyll *a*:*b* ratio			
Control	2.12 ± 0.03^a^	2.00 ± 0.01^a^	1.98 ± 0.03^a^
Strain*-* 1 (S_1_)	2.43 ± 0.04^b^	2.67 ± 0.02^bc^	2.88 ± 0.05^c^
Strain*-* 2 (S_2_)	2.88 ± 0.05^c^	2.93 ± 0.03^c^	3.00 ± 0.05^d^
Lime (L)	2.57 ± 0.05^b^	2.51 ± 0.04^b^	2.49 ± 0.04^b^
S_1_ + L	2.93 ± 0.06^c^	3.14 ± 0.04^d^	3.25 ± 0.06^de^
S_2_ + L	2.12 ± 0.03^a^	2.00 ± 0.01^a^	1.98 ± 0.03^a^

Mean values (mean ± SE, n = 4) followed by different letters are statistically different (ANOVA; Duncan’s multiple- test range, p ≤ 0.05).

#### Leaf nitrogen and chlorophyll content

**Figure 3:**
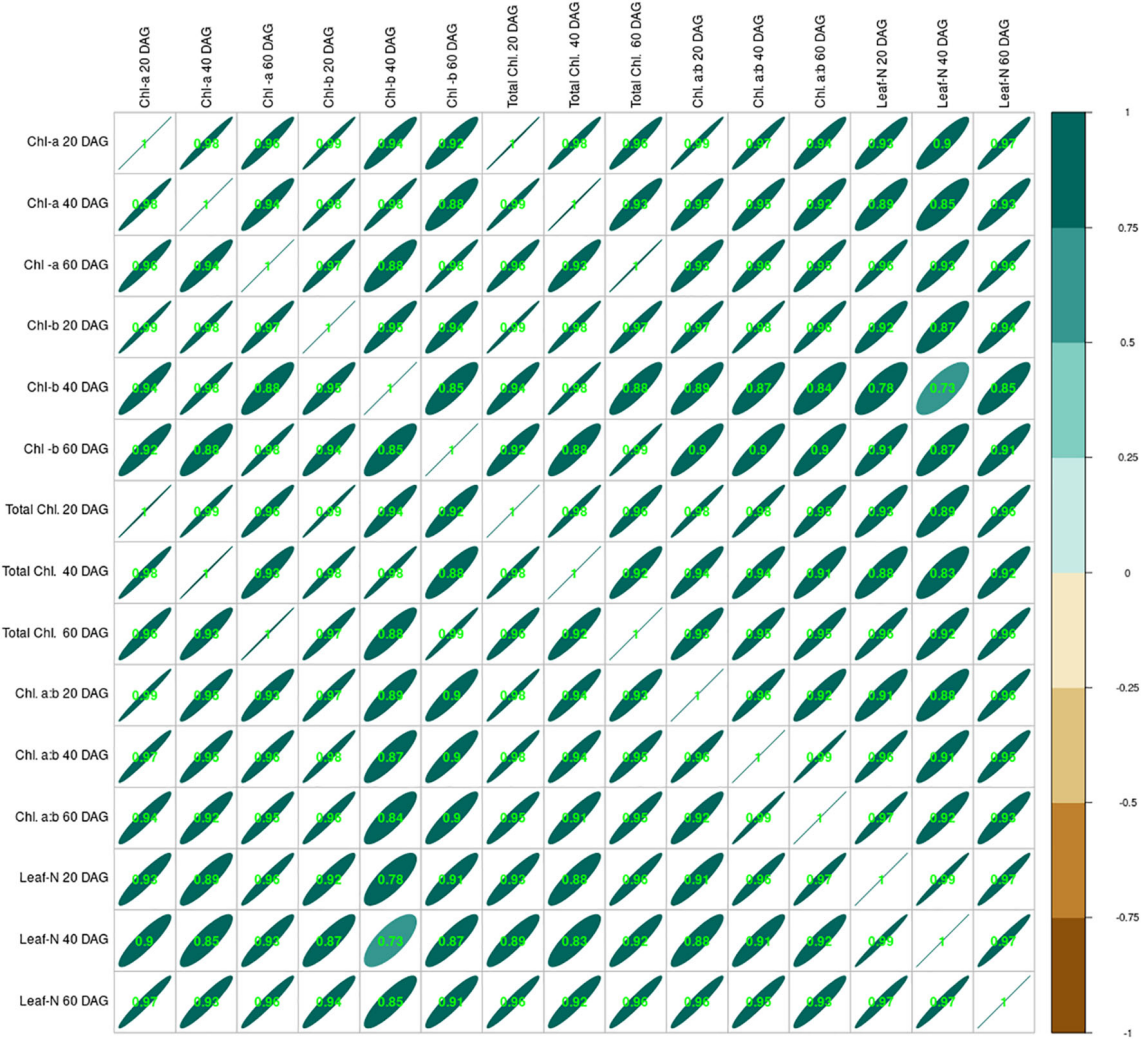
Correlation between leaf nitrogen content at 20, 40, and 60 DAG and chlorophyll (*a*, *b*, total and *a*:*b*) of French bean. DAG, days after germination.

Crop growth under the influence of seed inoculation with two isolated strains S1 and S2 and integration of lime application in acid soil influenced crop growth in many ways, including the leaf nitrogen content. Data relating to leaf nitrogen content are presented in **Table 2**. Leaf-N content ranged from 1.11% to 2.60%, 1.09% to 2.98%, and 0.98% to 3.21% at 20, 40, and 60 DAG, respectively. The lowest was measured in the control treatment, and the highest was in the S2 + L treatment. Leaf-N content increased from 20 to 60 DAG with native strain- inoculated treatments. There was a decrease in leaf-N content from 20 to 60 DAG in the control treatment, whereas with sole lime treatment, the leaf-N content was increased from 20 to 40 DAG and thereafter decreased. Seed inoculation with the S1 strain of *Rhizobium* alone increased the nitrogen content by 73%, but the leaf nitrogen could be increased to a 2.22% level by combining the practice of liming of acid soil, where it was 60% higher than the practice of lime alone. The S2 strain- inoculated crop continued to maintain higher nitrogen than the S1 inoculated crop even with or without liming practice. Leaf nitrogen content increased with the native strain inoculation except in control and lime-treated pots. The chlorophyll content of the leaf was increased throughout the growing stages, and chlorophyll content was positively correlated to the leaf nitrogen content (**Figure 3**).

**Table 2:** Influence of *Rhizobium* strains on leaf nitrogen content of French bean.

Treatments	Nitrogen (%)		
	Days after germination (DAG)		
	20	40	60
**Control**	1.11 ± 0.04^a^	1.09 ± 0.03^a^	0.98 ± 0.03^a^
**Strain*-* 1 (S_1_)**	1.92 ± 0.05^c^	2.17 ± 0.03^b^	2.29 ± 0.04^c^
**Strain*-* 2 (S_2_)**	2.26 ± 0.03^d^	2.35 ± 0.03^d^	2.49 ± 0.03^d^
**Lime (L)**	1.39 ± 0.04^b^	1.42 ± 0.03^a^	1.20 ± 0.03^b^
**S_1_ + L**	2.22 ± 0.03^d^	2.55 ± 0.04^c^	2.75 ± 0.02^d^
**S_2_ + L**	2.60 ± 0.04^e^	2.98 ± 0.04^e^	3.21 ± 0.03^e^

Mean values (mean ± SE, n = 4) followed by different letters are statistically different (ANOVA; Duncan’s multiple- test range, p ≤ 0.05).

#### Root characteristics

Data relating to root characteristics of French beans under the influence of seed inoculation with two native isolated strains under limed and unlimed conditions are presented in **Table 3**. At 20, 40, and 60 DAG, the root length of the rakia bean crop increased from 5.8 to 7.9, 6.7 to 8.1, and 7.9 to 9.4 cm, respectively. Seed inoculation with the S1 strain influenced the root length by 10% and the S2 strain by 17% compared to no seed inoculation practice control. Seed inoculation with *Rhizobium*, specifically with the S2 strain and liming practice, had a considerable influence on the root growth of the test crop. The root weight of French beans at 20, 40, and 60 DAG ranged from 2.4 to 4.4, 3.5 to 5.6, and 3.6 to 6.7 g plant ^−1^, respectively.

**Table 3:** Influence of *Rhizobium* strains on root characteristics of French bean crop.

Treatments	Days after germination (DAG)		
	20	40	60
Root length (cm)			
Control	5.8 ± 0.03^a^	6.7 ± 0.03^a^	7.9 ± 0.04^a^
Strain*-* 1 (S_1_)	6.4 ± 0.04^bc^	7.2 ± 0.04^ab^	8.4 ± 0.05^b^
Strain*-* 2 (S_2_)	6.8 ± 0.04^c^	7.6 ± 0.04^c^	8.9 ± 0.06^d^
Lime (L)	5.9 ± 0.05^b^	6.9 ± 0.04^a^	8.0 ± 0.05^a^
S_1_ + L	7.4 ± 0.06^d^	7.5 ± 0.05^bc^	8.7 ± 0.05^c^
S_2_ + L	7.9 ± 0.06^d^	8.1 ± 0.06^d^	9.4 ± 0.06^e^
Root biomass (g)			
Control	2.4 ± 0.01^a^	3.5 ± 0.02^a^	3.6 ± 0.01^a^
Strain*-* 1 (S_1_)	2.8 ± 0.01^b^	3.9 ± 0.02^b^	4.4 ± 0.02^b^
Strain*-* 2 (S_2_)	2.9 ± 0.02^b^	4.8 ± 0.03^c^	5.2 ± 0.02^cd^
Lime (L)	3.4 ± 0.03^c^	4.6 ± 0.03^c^	5.0 ± 0.02c
S_1_ + L	3.8 ± 0.03^d^	4.8 ± 0.03^c^	5.7 ± 0.03^d^
S_2_ + L	4.4 ± 0.04^e^	5.6 ± 0.05^d^	6.7 ± 0.04^e^
Root volume (m^3^)			
Control	5.1 ± 0.03^a^	5.3 ± 0.04^a^	5.6 ± 0.03^a^
Strain*-* 1 (S_1_)	5.8 ± 0.04^c^	6.1 ± 0.05^b^	6.3 ± 0.04^b^
Strain*-* 2 (S_2_)	5.9 ± 0.04^c^	6.4 ± 0.05^c^	6.8 ± 0.04^bc^
Lime (L)	5.3 ± 0.03^b^	6.1 ± 0.05^b^	6.6 ± 0.04^b^
S_1_ + L	5.7 ± 0.04^c^	6.8 ± 0.06^d^	7.1 ± 0.05^c^
S_2_ + L	6.0 ± 0.05^d^	6.9 ± 0.07^d^	7.8 ± 0.06^d^
Root density (g cm^−3^)			
Control	0.47 ± 0.01^a^	0.64 ± 0.01^a^	0.64 ± 0.01^a^
Strain*-* 1 (S_1_)	0.48 ± 0.01^b^	0.66 ± 0.01^b^	0.70 ± 0.02^b^
Strain*-* 2 (S_2_)	0.49 ± 0.01^b^	0.75 ± 0.02^d^	0.76 ± 0.01^c^
Lime (L)	0.64 ± 0.02^c^	0.75 ± 0.01^d^	0.76 ± 0.01^c^
S_1_ + L	0.67 ± 0.02^d^	0.71 ± 0.01^c^	0.80 ± 0.02^d^
S_2_ + L	0.73 ± 0.03^e^	0.81 ± 0.02^e^	0.86 ± 0.02^e^

Mean values (mean ± SE, n = 4) followed by different letters are statistically different (ANOVA; Duncan’s multiple- test range, p ≤ 0.05).

#### Nodular characteristics

**Figure 4:**
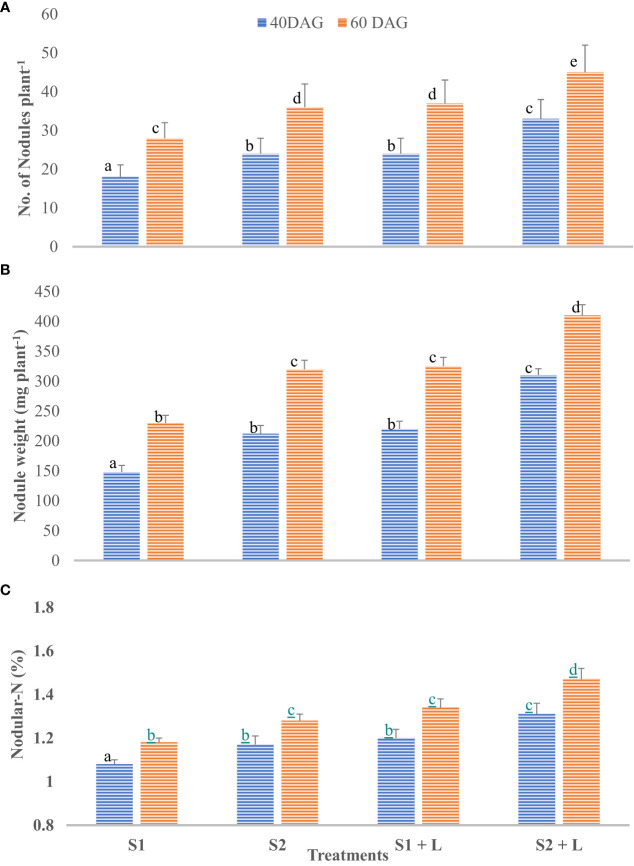
Influence of *Rhizobium* strains inoculation of seed on nodular characteristics of French bean. **(A)** Nodule number. **(B)** Nodule weight. **(C)** Nodular-N. T-shaped bar at the top of each bar indicates standard error (SE), and different letters on top indicate statistical difference (Duncan’s multiple-test range), p = 0.05.

Data related to the nodulation behavior of French beans after inoculation with isolated native strains are presented in **Figures 4A** **–C**. French bean plants did not bear nodules up to 20 days after germination in any of the five treatments tested. However, at 40 DAG, the nodules were observed in all the inoculated treatments, and their number and weight per plant increased to 60 DAG. This was corroborated by the findings of Khan et al. (1999). The S2 strain- inoculated plants possessed a significantly higher number and weight of nodules than did the S1 strain-inoculated plants. Integrating the practice of liming of acid soil with S1 strain inoculation of seeds increased the nodule number by 33% compared to no liming practice. The nodulation behavior of the crop at 40 days after germination followed a similar trend as it was at 60 DAG, with a difference that there was a considerable increase in the number. Liming of soil influenced the efficiency of the S1 strain by 11% and the S2 strain by 12% for nodular N content. Nodular N content increased considerably by 60 DAG and ranged from 1.18% to 1.47%. The extent of the increase was 9.0% to 12%. Soil amelioration with lime influenced N content ranging from 13.6% with S1 to 14.8% with the S2 strain. The S2 strain was a 9.5% better performer than the S1 strain concerning nodular N content. Not only the nodule number and their weight but also the nodular N were influenced by inoculation with two test strains of *Rhizobium*, and liming practice in acid soil improves molybdenum availability due to an increase in pH (Togay et al., 2008). Similarly, seed inoculation with both the test strains and liming of soil was observed to increase the root length, root volume, and root biomass of the French bean plants. Buerkert et al. (1990) reported that the lime application to acidic soil enhanced the nodular characteristics, and Bakari et al. (2020) reported that the soybean inoculation is effective in increasing nodulation and N fixation in moderately acidic soils, contrary to strongly acidic soils.3.2 Field experiment

The result of the pot experiment was verified with the help of a field experiment. The soil of the experimental site was sandy clay loam in texture, strongly acidic in reaction, high in organic carbon and available K, and low in available N, P, and S. The DTPA extractable Zn was adequate, and hot water extractable B was deficient (**Table 4**).

**Table 4:** Initial soil properties of the experimental site.

Parameters	Units	Values	Class/status
Textural class	–	Sand 62%, silt 16%, and clay 22%	Sandy clay loam
pHw (1:2.5)	–	4.68	Strongly acidic
Electrical conductivity (EC)	(dS m^−1^)	0.06	Normal
Organic carbon (OC)	(g kg^−1^)	1.50	High
Alkaline KMnO_4_ –N	(kg ha^−1^)	138	Low
Bray’s 1-P		8.00	Low
NH_4_OAc. K		313	High
CaCl_2_ extractable S		12.3	Low
DTPA-Zn	(mg kg^−1^ soil)	0.62	Adequate
Hot water extractable B		0.34	Deficient
Lime requirement to raise the pH to 6.5	(kg CaCO_3_ ha^−1^)	3530	**-**

Mean values (mean ± SE, n = 4) followed by different letters are statistically different (ANOVA; Duncan’s multiple- test range, p ≤ 0.05).

#### Productivity and relative agronomic efficiency

The productivity of the French bean crop under the influence of seed inoculation with an isolated local strain of *Rhizobium* and soil amelioration measures has been discussed in this section and shown in **Table 5**. Fresh pod yield of French bean under the influence of different packages of practices varied between 10.4 t ha^−1^ in control (contribution of soil alone) and 19.5 t ha^−1^ in the STD + S2 + L + B package. There was a significant influence of individual inputs used in this experiment in improving crop productivity. The use of two inoculants S1 and S2 increased the pod yield by 11% and 16%, respectively, compared to the control practice. Inoculants S1 and S2 increased the pod yield by 26% and 30% respectively, compared to the yield of 12.4 t ha^−1^ due to farmers’ practice. The yield of the French bean crop due to the farmers’ practice adopted was 29% less than the yield due to the STD practice, which was 12.4 t ha^−1^. Integration of STD practice with seed inoculation with the strains of *Rhizobium* increased the pod yield by 22% (S1 strain) and 25% (S2 strain) compared to STD alone. Combining the practice of liming of acid soil @ 0.2LR either with FP or with STD and seed inoculation with the S1 and S2 strains increased pod yield ranging from 33% to 39% compared to no liming practices. Supplementation of deficient micronutrient boron (applied @ 1 kg B ha^−1^) increased the pod yield considerably (14%–19%) and significantly compared to no- boron application.

**Table 5:** Effect of native *Rhizobium* strains with different nutrient management practices on yield and relative agronomic efficiency of French bean.

Treatments	Yield (t ha^−1^)	Response (%)	RAE (%)
Absolute Control	10.4 ± 0.03^a^	–	–
S_1_	11.5 ± 0.03^b^	11*	28
S_2_	11.9 ± 0.05^c^	16*	38
FP	12.4 ± 0.05^c^	(-)29^$^	50
FP + S_1_	14.5 ± 0.07^d^	26**	103
FP + S_2_	14.8 ± 0.08^d^	30**	110
FP + S_1_ + L	16.5 ± 0.08^e^	29***	153
FP + S_2_ + L	17.1 ± 0.09^e^	34****	168
STD	14.4 ± 0.09^de^	–	100
STD + S_1_	15.9 ± 0.11^e^	22^$$^	138
STD + S_2_	16.1 ± 0.12^f^	24^$$$^	143
STD + S_1_ + L	18.0 ± 0.13^g^	33^$$$^	190
STD + S_2_ + L	18.5 ± 0.15^h^	39^$$$^	203
STD + S_1_ + L + B	18.8 ± 0.15^h^	14^#^	210
STD + S_2_ + L + B	19.5 ± 0.16^i^	19^#^	228

Mean values (mean ± SE, n = 4) followed by different letters are statistically different (ANOVA; Duncan’s multiple- test range, p ≤ 0.05).

*Response compared to control.

**Response compared over FP.

***Response of lime over FP + S1.

****Response of lime over PF + S2 practice.

^$^Yield loss in FP compared to STD.

^$$^Response over STD.

^$$$^Response of lime over respective lime packages.

^#^Response of B application over respective non- B practice.

Relative agronomic efficiency (RAE) was calculated for different practices (**Table 5**) based on the pod yielding and efficiency of the soil test-based practice taken as 100. It varied widely between 28 and 228, with the lowest with seed inoculation with RBHR-15 (S1) alone and the highest with STD + L + B + S2 practice. Based on the RAE values, the different practices can be arranged in the following order: S1 (21)< S2 (52)< FP (55)< FP + S1 (84)< FP + S2 (93)<FP + L + S1 (127)< FP + L + S2 (138)< STD + S1 (147)< STD + S2 (180)< STD + L + S1 (203)< STD + L + B + S1 (233)< STD + L + S2 (234)< STD + L + B + S2 (264).

Pod yield of French beans varied significantly between 10.4 and 19.5 t ha^−1^, the lowest with farmers’ practice using imbalanced improper and insufficient fertilizers and the highest with fertilizer application based on soil tests correcting the deficient nutrient S and B along with seed inoculation with RBHR-21 strain of rhizobia. Farmers were losing 29% of the potential yield due to no adoption of soil test-based fertilizer application. Integration of seed inoculation with two locally isolated strains significantly increased the yields, particularly the S2 strain (30% with FP and 25% with STD) than the S1 strain (26% with FP and 16% with STD).

#### Farmers’ field demonstration

**Figure 5:**
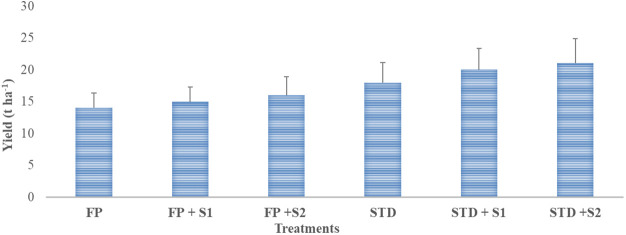
Influence of nutrient management practices on yield of French beans in farmers’ fields. T-shaped bar at the top of each bar indicates standard error (SE), and different letters on top indicate statistical difference (Duncan’s multiple-test range), p = 0.05.

Data related to pod yield under farmers’ field conditions are presented in **Figure 5**. The highest (21.0 t ha^−1^) yield was recorded in a package where strain RBHR-21 was inoculated with a soil test dose of fertilizer (STD + S2) followed by STD + S1, STD, FP + S2, and FP + S1, and the lowest (14.0 t ha^−1^) was recorded in farmers’ practice package. There was an increase in yield of 7% over farmers’ practice when strain RBHR-15 was inoculated alone; likewise, 14% with RBHR-21. Yield enhancement was 56% over the farmers’ practice when the soil test dose of fertilizer was applied, STD with RBHR-21 yield was 98%, and STD with RBHR-15 was 84% higher than farmers’ practice. There was the minimum benefit of the isolates on yield with STD, viz., 36% and 54% over STD with RBHR-15 and RBHR-21, respectively. Demonstrations indicated that both S1 (RBHR-15) and S2 (RBHR-21) were potential bioinoculants for the crop in the district.

## Discussion

Liming of acid soil influenced crop growth positively, particularly when the practice was integrated with seed inoculation with the S2 strain than the S1 strain because the liming neutralizes the acidity (Pattanayak and Sarkar, 2016; Pattanayak, 2016; Msimbira and Smith, 2020), which provides a better environment for the growth of native strain and activity. Native rhizobia strains produce the phytohormone indole-3-acetic acid (IAA), leading to better growth (Mehboob et al., 2012; Mabrouk et al., 2018; Nyaga and Njeru, 2020).

Liming of soil or its integration with seed inoculation with isolated strains increased chlorophyll content. Chlorophyll *b* content was less than that of chlorophyll *a* under the influence of various treatments in all the stages of growth. Input use resulted in an increase in total chlorophyll content throughout the growing period. A similar trend was also observed with chlorophyll *a* and chlorophyll *b*. The chlorophyll *a*:*b* ratio was higher in the native strain- inoculated treatments than in the control and only lime- applied treatments. The ratio of chlorophyll *a*:*b* increased in strain- inoculated pots from 20 DAG to 60 DAG except in control and sole lime- applied pots, where it was decreased. Inoculation of *Rhizobium* enhances the photosynthetic activity Elkoca et al. (2010).

Chlorophyll *a*, chlorophyll *b*, and total chlorophyll were increased at different stages of crop growth irrespective of the treatments. The chlorophyll *a*:*b* ratio shows a similar trend except for control and lime treatment (where it was decreased). This was attributed to the effects of efficient strains of microbial inoculants such as *Rhizobium* and *Bradyrhizobium* on the availability of nutrients in soils further by enhancing nutrient uptake by plants (Makoi et al., 2013; Tagore et al., 2014). The leaf nitrogen content positively influenced the leaf’s chlorophyll *a*, chlorophyll *b*, total chlorophyll, and chlorophyll *a*:*b* during crop growth. It was because nitrogen is a structural element of chlorophyll and protein, thereby affecting the formation of chloroplasts and the accumulation of chlorophyll in the leaf (Tucker, 2004 and Bassi et al., 2018).

Root weight increased throughout the growing period. The higher the root volume, the better the roots’ exposure in the rhizosphere is for nutrient absorption, crop establishment, and better productivity. Similar trends were observed with root volume and density. Liming of acid soil influenced the root characteristics, as it neutralizes the H^+^ and Al^3+^ (Grewal and Williams, 2003; Pattanayak, 2016), which were responsible for poor root growth. Similarly, Haling et al. (2010) reported that liming of acid soil increases root growth. Better root characteristics may change according to the abiotic and biotic stress, but better root condition in lime + *Rhizobium* treatment implies adequate availability of nutrients to the plants. Similarly, Strock et al. (2019) reported that the morphology and architecture are also influenced by different stress conditions, and a better one indicates adequate nutrient availability, which resulted in a higher seed yield of *P. vulgaris*.

Liming acid soil not only neutralizes the acidity due to H^+^ and Al^3+^ but also supplements the soil with Ca and Mg for crop nutrition. Liming practice improves soil aggregation, aeration, and water holding capacity, which ultimately favors root growth for nodulation, water, and nutrient uptake by the crop. Present findings are corroborated by the observations of Mishra et al. (2018). Seed inoculation with efficient *Rhizobium* strain supported by soil test-based fertilizer nutrient supplementation correcting the deficiencies (favoring balanced nutrition) and liming for neutralizing the soil acidity helped triple the French bean yield. The results of the present investigation are in agreement with the findings of earlier works on leguminous crops like soybean (Patra et al., 2012) and French bean (Biswas et al., 2020), green gram, black gram, red gram, and cowpea crops (Pattanayak and Rao, 2016).

## Conclusion

Both the isolated native strains RBHR-21 and RBHR-15 were efficient strains for nodulation and yield improvement of French beans. The RBHR-21 strain was better than RBHR-15 by 4%–14% in terms of contribution to the pod yield of French beans. The efficiency of strains can be increased with balanced fertilization of crops based on the soil test. Soil amelioration measure, namely, liming of acid soil @ 0.2 LR, improved the working efficiency of native isolated strains in terms of yield improvement ranging from 33% to 39%, with leaf N content of 46% to 72%. Amelioration of acid soil with lime and inoculation of native strains of rhizobia enhanced the root characteristic comparison of non-limed packages. Supplementation of deficient micronutrients (B) also had a positive impact on yield improvement ranging from 14% to 19%. Integration of input use like seed inoculation with efficient strains of *Rhizobium*, maintaining optimum growing conditions of the soil in terms of balanced nutrition through soil test-based fertilization, soil amelioration measure by liming of acid soil, and supplementation of deficient micronutrient(s) were the best approaches in yield maximization of French bean.

## Data availability statement

The original contributions presented in the study are included in the article/supplementary material. Further inquiries can be directed to the corresponding author.

## Author contributions

PA, RP, and SKP conceived the ideas and designed methodology; PA, DS, SKM collected the data; PA, DS and NP analyzed the data; DS, RP, KP, SS, TS, SM and SP led the writing of the manuscript. All authors contributed critically to the drafts and gave final approval for publication.
